# *Piper sarmentosum* Roxb. Inhibits Angiotensin-Converting Enzyme Activity in Phorbol 12-Myristate-13-Acetate-Induced Endothelial Cells

**DOI:** 10.3390/ijms25052806

**Published:** 2024-02-28

**Authors:** Azizah Ugusman, Siti Marjiana Ismail, Nur Syahidah Nor Hisam, Chua Kien Hui, Mohammed S. M. Saleh, Abdul Kadir Abdul Karim, Nur Syakirah Othman, Adila A. Hamid, Amilia Aminuddin

**Affiliations:** 1 Department of Physiology, Faculty of Medicine, Universiti Kebangsaan Malaysia, Kuala Lumpur 56000, Malaysia; marjiana@um.edu.my (S.M.I.); p104164@siswa.ukm.edu.my (N.S.N.H.); ckienhui@ppukm.ukm.edu.my (C.K.H.); p118667@siswa.ukm.edu.my (N.S.O.); adilahamid@ppukm.ukm.edu.my (A.A.H.); 2 Mushroom Research Centre, Institute of Biological Sciences, Faculty of Science, University of Malaya, Kuala Lumpur 50603, Malaysia; 3 Programme of Biomedical Science, Centre for Toxicology & Health Risk Studies, Faculty of Health Sciences, Universiti Kebangsaan Malaysia, Kuala Lumpur 50300, Malaysia; 4 Department of Pharmacology, Faculty of Medicine, Universiti Kebangsaan Malaysia, Kuala Lumpur 56000, Malaysia; ksm20085@hotmail.com; 5 Department of Obstetrics and Gynaecology, Faculty of Medicine, Universiti Kebangsaan Malaysia, Kuala Lumpur 56000, Malaysia; abdulkadirabdulkarim@yahoo.com

**Keywords:** angiotensin converting enzyme, hypertension, human umbilical vein endothelial cells, phorbol 12-myristate-13-acetate, *Piper sarmentosum*

## Abstract

Angiotensin-converting enzyme (ACE) plays a crucial role in the pathogenesis of hypertension. *Piper sarmentosum* Roxb., an herb known for its antihypertensive effect, lacks a comprehensive understanding of the mechanism underlying its antihypertensive action. This study aimed to elucidate the antihypertensive mechanism of aqueous extract of *P. sarmentosum* leaves (AEPS) via its modulation of the ACE pathway in phorbol 12-myristate-13-acetate (PMA)-induced human umbilical vein endothelial cells (HUVECs). HUVECs were divided into five groups: control, treatment with 200 µg/mL AEPS, induction 200 nM PMA, concomitant treatment with 200 nM PMA and 200 µg/mL AEPS, and treatment with 200 nM PMA and 0.06 μM captopril. Subsequently, ACE mRNA expression, protein level and activity, angiotensin II (Ang II) levels, and angiotensin II type 1 receptor (AT1R) and angiotensin II type 2 receptor (AT2R) mRNA expression in HUVECs were determined. AEPS successfully inhibited ACE mRNA expression, protein and activity, and angiotensin II levels in PMA-induced HUVECs. Additionally, AT1R expression was downregulated, whereas AT2R expression was upregulated. In conclusion, AEPS reduces the levels of ACE mRNA, protein and activity, Ang II, and AT1R expression in PMA-induced HUVECs. Thus, AEPS has the potential to be developed as an ACE inhibitor in the future.

## 1. Introduction

Cardiovascular diseases (CVD) are the number one cause of death worldwide, with an estimated 17.9 million deaths in 2019 [1]. Hypertension, physical inactivity, smoking, dyslipidemia, and diabetes mellitus are major modifiable risk factors for CVD. However, hypertension is the main contributor to CVD morbidity and mortality [2]. The pathogenesis of primary hypertension involves several mechanisms, such as oxidative stress [3] and overactivity of the renin–angiotensin system (RAS) [4].

RAS regulates blood pressure and body fluid homeostasis and is a key therapeutic target in hypertension [5]. There are several components in RAS, namely, renin, angiotensinogen, angiotensin-converting enzyme (ACE), angiotensin I (Ang I), and angiotensin II (Ang II). Studies have demonstrated that the local RAS components are also found in the endothelium, which plays a fundamental role in the regulation of vasomotor tone and oxidative metabolism [6].

One of the most crucial components of RAS is the ACE, which can be found in the endothelial cells lining the vascular wall. Overexpression of the ACE gene resulted in exaggerated ACE biosynthesis, increased ACE activity, and hypertension development [7]. ACE is primarily known for its ability to cleave Ang I to form Ang II, a major bioactive peptide. The actions of Ang II are mediated by its binding to the angiotensin II type 1 receptor (AT1R) and the angiotensin II type 2 receptor (AT2R) [8].

The binding of Ang II to AT1R causes vasoconstriction, increased blood pressure, and several other adverse effects, such as oxidative stress and inflammation. Hence, inhibition of ACE reduces the formation of Ang II, which in turn lowers blood pressure [9]. In addition, inhibition of ACE might increase the binding of Ang II to AT2R, which causes vasodilation and decreases blood pressure [10]. AT2R is abundant during fetal life, but its expression decreases markedly in the postnatal period. Despite the low expression of AT2R in adults, the binding of Ang II to AT2R leads to vasodilation and decreases blood pressure [10]. However, most of the actions of Ang II are mediated via its binding to AT1R [11].

ACE inhibition is the most common test used to screen for the antihypertensive activity of a substance in vitro [12]. Meanwhile, phorbol 12-myristate 13-acetate (PMA) is routinely used to induce ACE activity in vitro and in vivo [13]. PMA is a polyfunctional diterpene phorbol ester isolated from *Croton tiglium* and a protein kinase C (PKC) activator that has been widely used as an ACE inducer. In addition, PMA mimics the action of diacylglycerol, which is an endogenous PKC activator [14]. PKC activation increases ACE gene transcription and secretion in human umbilical vein endothelial cells (HUVEC). Furthermore, PKC activation is involved in the development of hypertension [15].

ACE inhibition is a primary target for hypertensive therapy. ACE inhibitors, such as captopril, enalapril, and perindopril, are routinely prescribed to treat hypertension. However, the side effects of synthetic ACE inhibitors, such as cough, angioedema, renal failure, and harmful effects in pregnancy, have been reported [16]. Hence, it is important to explore natural sources of ACE inhibitors with potentially fewer side effects for the prevention and complementary treatment of hypertension. In phytomedicine, different types of naturally occurring flavonoids, such as anthocyanins, flavones, flavonols, and flavanols, exhibit ACE inhibitory activity [17].

*Piper sarmentosum* Roxb. (PS) is an herbaceous plant that belongs to the Piperaceae family and is widely distributed in the tropical and subtropical regions of the world [18]. Traditionally, *P. sarmentosum* is used to treat many diseases, such as diabetes, hypertension, and joint aches [19]. Toxicity studies have shown that the aqueous extract of *P. sarmentosum* leaves (AEPS) is safe to consume [20]. AEPS contains active compounds such as alkaloids [21], amides [22], pyrones [23], and flavonoids [24].

Furthermore, AEPS exhibits multiple cardiovascular protective effects, such as antidiabetic [19], antioxidant [25], anti-inflammatory [26], and anti-atherosclerotic effects [27]. The antihypertensive effect of AEPS has been validated in various hypertensive animal models, including spontaneously hypertensive rats [28,29,30], dexamethasone-induced hypertensive rats [31,32], and L-NAME-induced hypertensive rats [33]. A meta-analysis has demonstrated that AEPS supplementation significantly lowers systolic blood pressure, diastolic blood pressure and mean arterial pressure in hypertensive rat models [19]. Despite the confirmed in vivo antihypertensive effect of AEPS, the underlying mechanism, particularly its effect on the ACE pathway, is yet to be established. Therefore, the primary focus of this study was to investigate the effect of AEPS on the ACE pathway in PMA-induced endothelial cells, with the aim of elucidating the antihypertensive mechanisms of AEPS.

## 2. Results

### 2.1. LC-MS Analysis of AEPS

**Figure 1:**
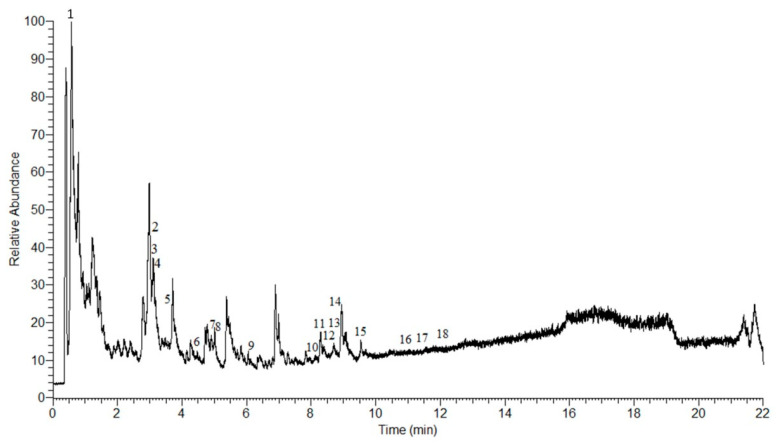
The LC-MS analysis of aqueous extract of *P. sarmentosum* leaves. Peaks are identified with numbers according to the elution order (refer to Table 1).

In this study, 18 compounds were detected in AEPS using Q Exactive HF Orbitrap mass spectrometry (Figure 1). The compounds were characterized using retention times (Rt) and mass spectra, as listed in Table 1. Among these 18 compounds, there are 12 alkaloids (cenocladamide, pipermethystine, N-(3-phenylpropanoyl) pyrrole, 1-[7-(3,4-methylenedioxyphenyl)-2,6-heptadienoyl] pyrrolidine, piperine, piperlonguminine, awaine, piplartine, peepuloidine, cepharadione A, sarmentine, and 1-[9-(3,4-methylenedioxyphenyl)-2,4,8-nonatrienoyl] pyrrolidine), three amides (sarmentamide A, sarmentamide B, and retrofractamide), one flavonoid (quercetin 3-rhamninoside), one organic acid (piperonylic acid) and one hydrazone (2,4,5-trimethoxybenzaldehyde hydrazone N-oxide).

### 2.2. Determination of the Optimum Concentrations of PMA, AEPS, and Captopril for HUVEC Treatments

**Figure 2:**
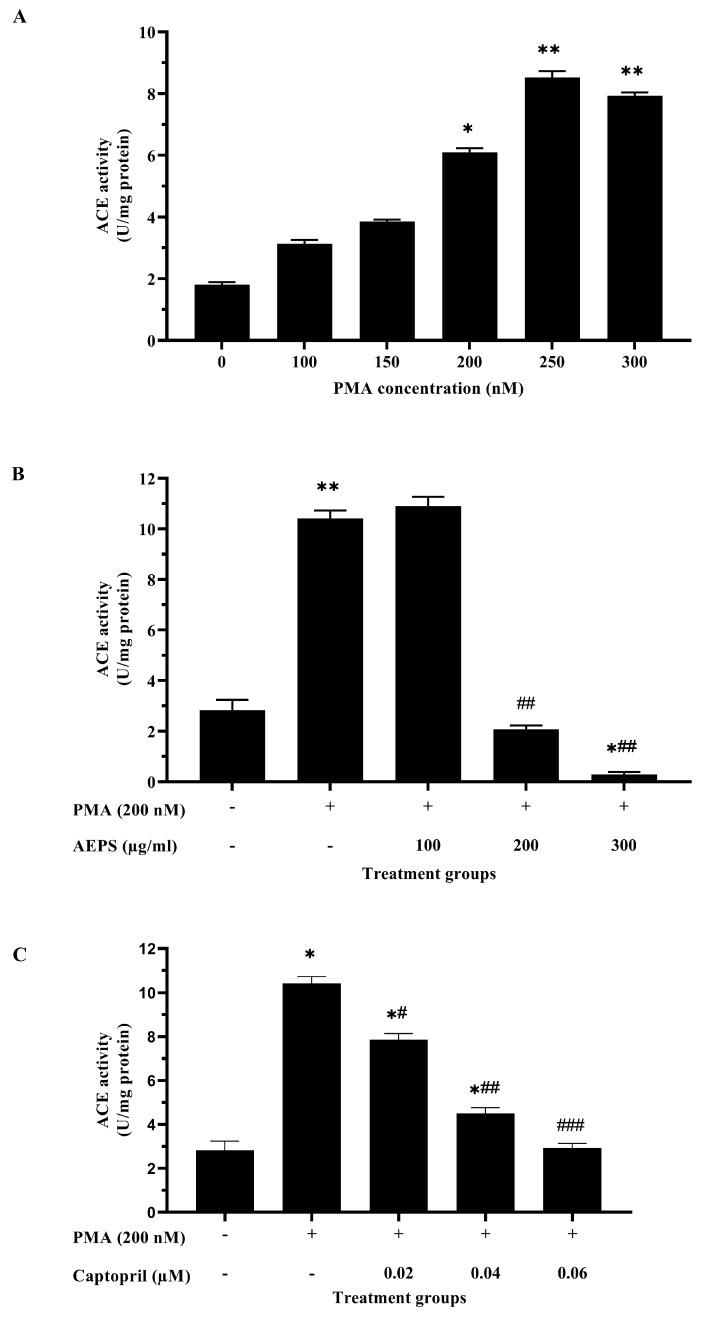
Determination of the optimum concentrations of PMA, AEPS, and captopril for HUVEC treatments based on ACE activity. (**A**) ACE activity in HUVECs induced with 100–300 nM PMA, (**B**) ACE activity in PMA-induced HUVECs upon treatment with different concentrations of AEPS (100–300 µg/mL), and (**C**) ACE activity in PMA-induced HUVECs upon treatment with different concentrations of captopril (0.02–0.06 µM). Values are expressed as mean ± SEM, n = 6. * *p* < 0.05, ** *p* < 0.01 compared with the untreated control, # *p* < 0.05, ## *p* < 0.01, ### *p* < 0.001 compared with the PMA group. ACE, angiotensin-converting enzyme; AEPS, aqueous extract of *Piper sarmentosum* leaves; PMA, phorbol 12-myristate-13-acetate.

A preliminary study to identify the optimal concentrations of PMA, AEPS, and captopril was conducted; hence, an effective treatment could be applied to the cells. ACE activity was increased in HUVECs exposed to 200 nM PMA (*p* < 0.05), 250 nM PMA (*p* < 0.01), and 300 nM PMA (*p* < 0.01) (Figure 2A). As the first concentration that significantly increased ACE activity in HUVECs, 200 nM PMA was chosen to induce ACE activity in subsequent experiments. Meanwhile, treatment of PMA-induced HUVECs with 200 μg/mL and 300 μg/mL AEPS successfully inhibited ACE activity (*p* < 0.01) (Figure 2B). However, there was no significant difference between the effects of 200 µg/mL and 300 µg/mL AEPS on ACE activity in HUVECs. Therefore, 200 μg/mL AEPS was used as the optimum concentration for subsequent assays. In addition, captopril was used as a positive control. ACE activity was inhibited in PMA-induced HUVECs treated with 0.02 µM (*p* < 0.05), 0.04 µM (*p* < 0.01), and 0.06 µM captopril (*p* < 0.001) (Figure 2C). Nevertheless, ACE activity in PMA-induced HUVECs treated with 0.02 µM and 0.04 µM captopril was still higher than that in the negative control group (*p* < 0.05). Only treatment with 0.06 µM captopril managed to decrease ACE activity to a level comparable to that in the negative control group. Therefore, 0.06 µM captopril was used as the optimum concentration in subsequent experiments.

### 2.3. Effect of AEPS on ACE mRNA Expression and Protein Levels in PMA-Induced HUVEC

**Figure 3:**
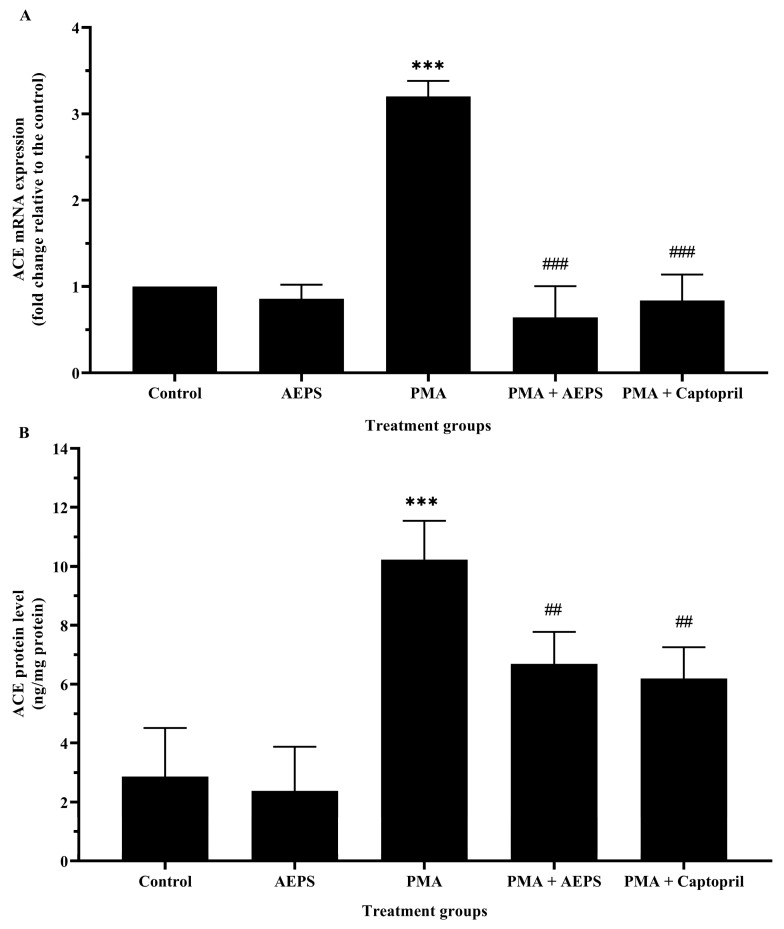
Effects of AEPS treatment on (**A**) ACE mRNA expression and (**B**) ACE protein levels in HUVECs induced with PMA. Values are expressed as mean ± SEM, n = 6. *** *p* < 0.001 compared with control, ## *p* < 0.01, ### *p* < 0.001 compared with PMA group. ACE, angiotensin-converting enzyme; AEPS, aqueous extract of *Piper sarmentosum* leaves; PMA, phorbol 12-myristate-13-acetate.

Treatment with AEPS alone had no significant effect on ACE mRNA expression and protein levels compared to the control group (Figure 3). In the PMA group, ACE mRNA expression was upregulated by 3.20-fold compared with the control group (*p* < 0.001). Consequently, HUVECs treated with PMA also demonstrated higher ACE protein levels compared with the control group (*p* < 0.001). Treatment of PMA-induced HUVECs with AEPS attenuated ACE mRNA expression (*p* < 0.001) and protein levels (*p* < 0.01). Similarly, treatment with captopril reduced ACE mRNA expression (*p* < 0.001) and protein levels (*p* < 0.01) compared with the PMA group. There was no significant difference in the mRNA expression and protein levels of ACE between the AEPS and captopril groups.

### 2.4. Effect of AEPS on ACE mRNA Expression and Protein Levels in PMA-Induced HUVEC

**Figure 4:**
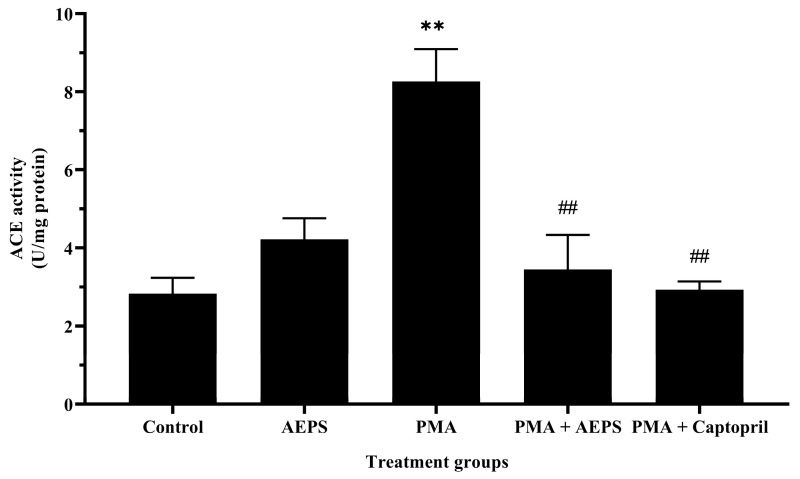
Effects of AEPS treatment on ACE activity in PMA-induced HUVECs. Values are expressed as mean ± SEM, n = 6. ** *p* < 0.01 compared with control. ## *p* < 0.01 compared with PMA group. ACE, angiotensin-converting enzyme; AEPS, aqueous extract of *Piper sarmentosum* leaves; PMA, phorbol 12-myristate-13-acetate.

Treatment with AEPS alone had no significant effect on ACE activity compared with the control group (Figure 4). PMA induction stimulated ACE activity compared with the control group (*p* < 0.01). Treatment of PMA-induced HUVECs with AEPS successfully inhibited ACE activity (*p* < 0.01). Similarly, treatment with captopril attenuated ACE activity (*p* < 0.01) compared with the PMA group. There was no significant difference in ACE activity between the AEPS and captopril groups.

### 2.5. Effect of AEPS on Angiotensin II Protein Levels in PMA-Induced HUVEC

**Figure 5:**
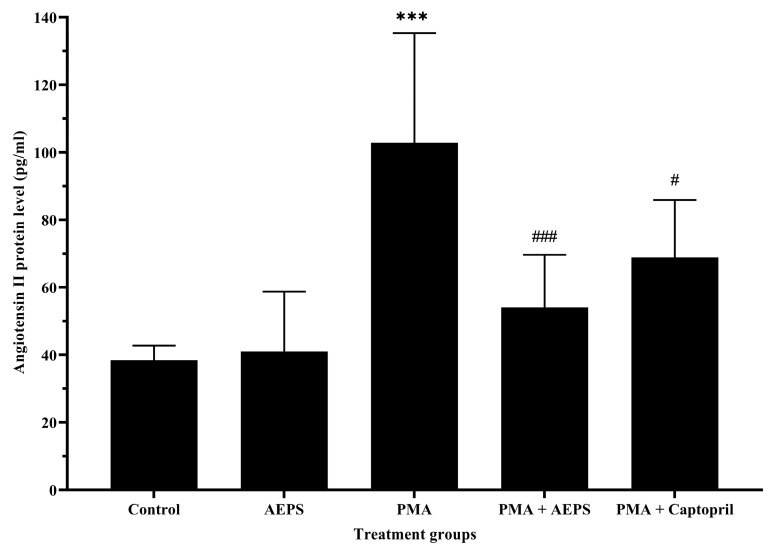
Effects of AEPS treatment on angiotensin II protein levels in PMA-induced HUVEC. Values are expressed as mean ± SEM, n = 6. *** *p* < 0.001 compared with control. # *p* < 0.05, ### *p* < 0.001 compared with PMA group. ACE, angiotensin-converting enzyme; AEPS, aqueous extract of *Piper sarmentosum* leaves; PMA, phorbol 12-myristate-13-acetate.

Treatment with AEPS alone had no significant effect on Ang II protein levels compared with the control group (Figure 5). Induction with PMA increased Ang II levels compared with the control group (*p* < 0.001). The increase in Ang II levels following PMA induction was successfully attenuated by AEPS (*p* < 0.001) and captopril (*p* < 0.05) treatment. There was no significant difference in Ang II levels between the AEPS and captopril groups.

### 2.6. Effect of AEPS on AT1R and AT2R mRNA Expression in PMA-Induced HUVEC

**Figure 6:**
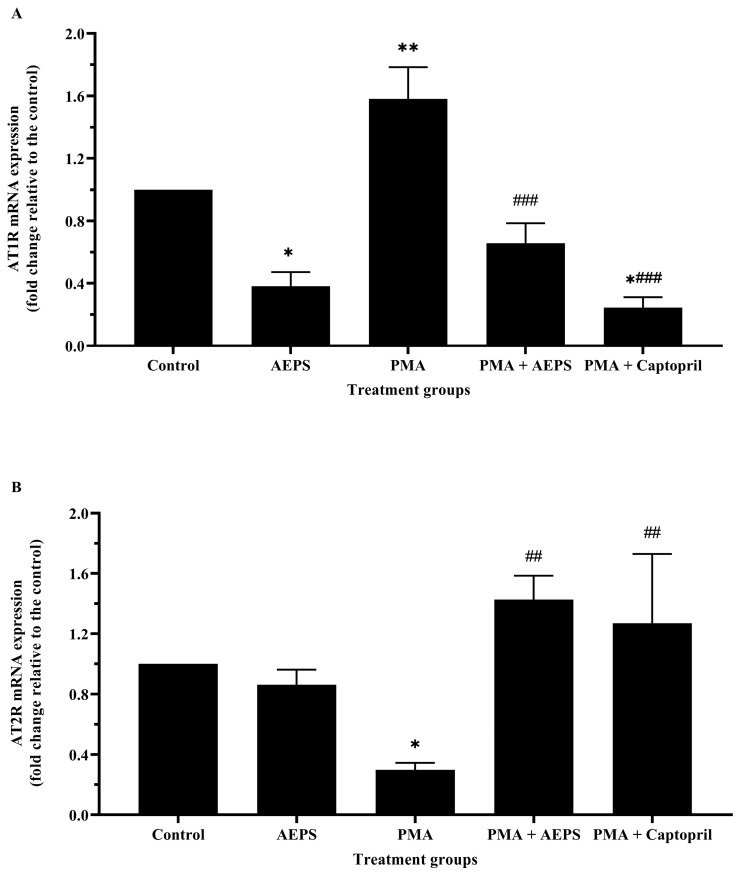
Effect of AEPS on (**A**) AT1R and (**B**) AT2R mRNA expressions in PMA-induced HUVEC. Values are expressed as mean ± SEM, n = 6. * *p* < 0.05, ** *p* < 0.01 compared with control. ## *p* < 0.01, ### *p* < 0.001 compared with PMA group. ACE, angiotensin-converting enzyme; AEPS, aqueous extract of *Piper sarmentosum* leaves; AT1R, angiotensin II type 1 receptor; AT2R, angiotensin II type 2 receptor; PMA, phorbol 12-myristate-13-acetate.

Treatment with AEPS alone reduced AT1R mRNA expression (*p* < 0.05) and had no significant effect on AT2R mRNA expression compared with the control group (Figure 6). In the PMA group, AT1R mRNA expression was upregulated 1.58-fold (*p* < 0.01), whereas AT2R mRNA expression was downregulated 0.29-fold (*p* < 0.05) compared with the control group. Treatment of PMA-induced HUVECs with AEPS downregulated AT1R mRNA expression (*p* < 0.001) and upregulated AT2R mRNA expression (*p* < 0.01). Similarly, treatment with captopril also reduced AT1R mRNA expression (*p* < 0.001) and increased AT2R mRNA expression (*p* < 0.01) compared with the PMA group. There was no significant difference in the mRNA expression of AT1R and AT2R between the AEPS and captopril groups.

### 2.7. Effect of AEPS on Ex Vivo Aortic Contraction to PMA

**Figure 7:**
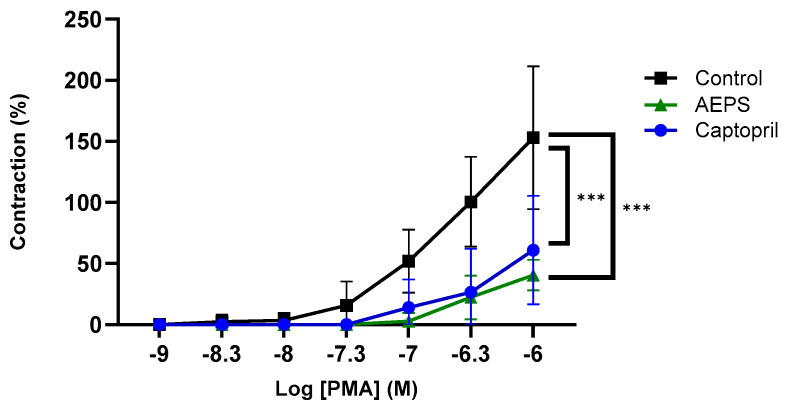
Effect of AEPS on ex vivo aortic contraction to PMA. Values are expressed as mean ± SEM, n = 6. *** *p* < 0.001 compared with control. AEPS, aqueous extract of *Piper sarmentosum* leaves; PMA, phorbol 12-myristate-13-acetate.

Figure 7 shows the contraction response of aortic rings to PMA at concentrations ranging from 10^−9^–10^−6^ M. There was a significant decrease in the contraction response to PMA in aortic rings pretreated with AEPS compared with the untreated control (*p* < 0.001). Pretreatment with captopril also decreased the aortic contraction response compared with the untreated control (*p* < 0.001). Meanwhile, there was no significant difference in the aortic contraction between the AEPS and captopril groups.

## 3. Discussion

In this study, we explored the mechanisms underlying the antihypertensive activity of AEPS, focusing on its ACE-inhibitory effects in PMA-induced HUVECs. We found that AEPS successfully reduced ACE mRNA expression, ACE protein levels, ACE activity, Ang II levels, and AT1R mRNA expression and increased AT2R mRNA expression in PMA-induced HUVEC. The in vitro experiment findings in PMA-induced HUVECs were further validated in an ex vivo experiment. In this case, pretreatment of aortic rings with AEPS resulted in a reduced contractile response to PMA.

Treatment with AEPS alone was conducted to investigate its regulation of ACE activity in control HUVECs. We observed insignificant changes in ACE mRNA expression, ACE protein levels, ACE activity, Ang II levels, and AT2R mRNA expression in HUVECs treated with AEPS alone compared with the control group. These results suggest that AEPS did not exhibit any inhibitory or inductive effects on the ACE pathway in the healthy state.

Meanwhile, the induction of HUVECs with PMA increased the levels of ACE mRNA, ACE protein, ACE activity, and Ang II. This is consistent with a previous study, which showed that HUVECs exposed to 250 nM PMA for 36 h exhibited higher ACE activity than the control group [34]. Additionally, another study also found a five-fold increase in ACE activity when HUVECs were induced with 100 ng/mL PMA for 24 h [35]. In addition to stimulating ACE activity, PMA also increased ACE mRNA expression through PKC activation [35]. The activation of PKC stimulates extracellular signal-regulated kinase (ERK) 1/2, which phosphorylates the ternary complex factor (TCF). TCF then binds to the early growth response gene-1 (EGR-1), subsequently stimulating EGR-1 expression [36]. ERK 1/2 also plays a role in phosphorylating and activating c-jun, leading to the formation of activator protein-1 (AP-1). Both EGR-1 and AP-1 are involved in the transcriptional activation of the ACE gene in PMA-induced human endothelial cells [36]. In short, PMA activates PKC, which stimulates ACE gene transcription, secretion, and activity in HUVEC.

The main function of ACE is to convert Ang I to Ang II, which binds to AT1R or AT2R to exert physiological effects [37]. The results of this study demonstrated that PMA-induced HUVEC exhibited high levels of Ang II and AT1R mRNA expression, whereas AT2R mRNA expression was low. Since PMA stimulates ACE activity, there is an increased production of Ang II by HUVECs. The increased expression of AT1R, compared with AT2R, results in greater binding of Ang II to AT1R. This binding triggers a vasoconstriction response and an increase in blood pressure [38]. A previous study also showed that increased levels of Ang II lead to an upregulation of AT1R mRNA expression [39].

Conversely, AEPS was able to downregulate ACE mRNA expression in HUVECs induced with PMA. This resulted in less ACE protein being synthesized, and subsequently, ACE activity was reduced. The inhibition of AEPS on ACE activity leads to less conversion of Ang I to Ang II. In addition to lowering Ang II levels, AEPS also downregulated the expression of AT1R, whereas AT2R expression was upregulated. The generated Ang II bound more to AT2R because of its higher expression compared with AT1R. The binding of Ang II to AT2R results in positive effects, such as vasodilation and increased synthesis of nitric oxide (NO), which helps lower blood pressure [11]. However, in this study, we did not measure the effect of AEPS on endothelial NO and its major producer, endothelial nitric oxide synthase (eNOS). Nevertheless, previous studies have demonstrated that AEPS stimulates eNOS expression and activity, as well as NO production in HUVECs exposed to oxidative stress and inflammatory stimuli [40].

Together, our findings indicate that AEPS exerted its antihypertensive effect through its modulation of ACE, AT1R, and AT2R. Interestingly, the modulatory effects of AEPS on ACE, AT1R, and AT2R were comparable to those of the conventional ACE inhibitor, captopril. This indicates that AEPS has great potential as a natural ACE inhibitor for treating hypertension. In this study, captopril demonstrated the ability to reduce ACE mRNA, protein and activity, Ang II levels, and AT1R expression while concurrently increasing AT2R expression. Captopril, a commonly prescribed ACE inhibitor, interacts with the zinc ion in the active site of ACE, hindering the enzyme’s ability to catalyze the conversion of Ang I to Ang II [41]. Consequently, there was a reduction in the levels of Ang II. Captopril also decreased ACE mRNA expression, leading to a subsequent reduction in ACE protein levels and activity. This collective action results in an overall decrease in Ang II production. The simultaneous downregulation of AT1R and upregulation of AT2R play additional roles in enhancing the antihypertensive effects of captopril.

Based on the LC-MS analysis, one of the active compounds present in AEPS was quercetin 3-rhamninoside. Quercetin 3-rhamninoside isolated from the extract of *Actinidia macrosperma* demonstrated an ACE inhibitory effect [42]. In addition, the presence of piperine in AEPS may also contribute to its ACE inhibitory effect. Piperine isolated from *Piper longum* L. fruits has been shown to have an antihypertensive effect through ACE inhibition [43]. Inhibition of ACE by piperine contributes to its vasorelaxant and hypotensive effects [44,45]. In addition, a previous study showed the presence of quercetin and rutin in AEPS [46]. Quercetin and rutin have been proven to have blood pressure-lowering effects [47,48]. One of the underlying mechanisms of the antihypertensive effects of quercetin and rutin is ACE inhibition [48,49]. However, quercetin’s bioavailability is generally considered low, primarily due to factors such as its poor solubility, limited absorption in the gastrointestinal tract, and rapid metabolism [50]. Several strategies have been explored to enhance quercetin’s bioavailability. These strategies often involve the complexation of quercetin with other molecules or encapsulation within delivery systems like cyclodextrins, liposomes, or polymers to improve its stability and absorption [51]. Improving bioavailability is important as it enhances the effectiveness of quercetin in exerting its potential health benefits, including its antihypertensive properties.

Since this study utilized a crude extract of *P. sarmentosum* rather than its purified active compound, it was not possible to pinpoint the specific component of the extract responsible for the observed effects. Nevertheless, it is proposed that the effects were probably attributed to the aforementioned active compounds. A placebo-controlled clinical trial is also necessary before incorporating AEPS as a viable antihypertensive supplement. Conducting such a study in a clinical setting would allow for an investigation into the optimal dosage, as well as an exploration of the pharmacokinetics and pharmacodynamics of AEPS.

## 4. Materials and Methods

### 4.1. Aqueous Extract of P. sarmentosum Leaf Preparation

Fresh leaves of *P. sarmentosum* were obtained from the Ethno Resources farm in Sungai Buloh, Malaysia. The leaves were identified by a plant taxonomist at the Forest Research Institute Malaysia (specimen voucher number FRI 45870). The preparation of AEPS was performed according to the method described previously [30]. Fresh leaves were cut into small pieces, sun-dried, and ground into powder form. The powder was then mixed with water (10%, *w*/*v*) and boiled at 80 °C for 3 h in a reflux extractor. The extract was subsequently filtered and dried using the freeze-drying method. The AEPS powder was then stored at 4 °C until use.

### 4.2. Liquid Chromatography (LC)-Mass Spectrometry (MS) Analysis of AEPS

LC-MS analysis was performed to identify the compounds in AEPS. LC was conducted using the Dionex UltiMate 3000 ultra-high-performance liquid chromatography (UHPLC) system (Thermo Fisher Scientific, Waltham, MA, USA) coupled with a Thermo Syncronis C18 column (1.7 µm × 100 mm × 2.1 mm; Thermo Fisher Scientific, Waltham, MA, USA). The column temperature was maintained at 55 °C, with an injection volume of 2 µL and a flow rate of 450 µL/min. The mobile phases comprised solvent A (water with the addition of 0.1% formic acid) and solvent B (acetonitrile with 0.1% formic acid). The gradient elution protocol started with 0.5% of solvent B for 1 min, followed by a linear increase from 0.5% to 99.5% of solvent B over 15 min, and was maintained for 4 min. Subsequently, the column was conditioned as initial and stabilized for 2 min before the next injection. MS data were obtained using a Thermo Scientific Q Exactive HF Orbitrap mass spectrometry system (Thermo Fisher Scientific, Waltham, MA, USA). The mass range for detection was set between 100 and 1000 *m*/*z*, and the stepped normalized collision energy was 20, 40, and 60 arbitrary units. The mass spectrometer operated with an MS1 resolution of 60,000 and an MS2 resolution of 15,000. Positive and negative heated electrospray ionization (HESI) were deployed at 3.9 and 3.3 kV, respectively. The ion source conditions were set as follows: capillary temperature of 320 °C, sheath gas flow rate of 28, aux gas flow rate of 8, sweep gas flow rate of 0, and aux gas heater temperature of 320 °C. All data were processed with the Xcalibur™ 4.0 software (Thermo Fisher Scientific, Waltham, MA, USA).

### 4.3. HUVEC Isolation and Culture

This study was approved by the Ethical Research Committee of Universiti Kebangsaan Malaysia (approval number: FF-2017-040). Human umbilical cords were collected after obtaining informed consent from healthy subjects in the labor room of Hospital Universiti Kebangsaan Malaysia. HUVECs were isolated from human umbilical cords via the collagenase perfusion technique as described previously [31]. Briefly, the cells were isolated from umbilical cords using 0.1% collagenase (Worthington Biochemical Corporation, Lakewood, NJ, USA) and cultured in endothelial cell medium (ScienCell Research Laboratories, Inc., San Diego, CA, USA) at 37 °C in a humidified atmosphere of 5% CO_2_ and 95% air. HUVECs at passage 3 at 80% confluency were used for all experiments.

### 4.4. Measurement of ACE Activity

The optimal doses of PMA, AEPS, and captopril for HUVEC treatments were determined on the basis of their effects on ACE activity. In preliminary experiments, HUVECs were treated with different concentrations of PMA (100–300 nM), AEPS (100–300 µg/mL), or captopril (0.02–0.06 µM) for 24 h. ACE activity in HUVECs was then measured using a colorimetric assay as described previously [32]. The principle of the assay is based on the ability of ACE to hydrolyze the synthetic substrate, hippuryl-histidine-leucine (HHL). A total of 200 µL HHL substrate solution was added to 200 µL HUVEC lysates. The mixture was incubated at 37 °C for 15 min. The enzyme reaction was stopped by adding 250 µL of hydrochloric acid. Ethyl acetate was then added, and the mixture was centrifuged. The resultant organic solution was separated into a glass tube and dried at 40 °C. Then, acetic anhydride and p-dimethylamino-benzaldehyde solutions were added, and the mixture was incubated at 40 °C for 40 min. The absorbance of the samples was measured using a colorimetric microplate reader at 490 nm wavelength. Based on the results of the preliminary experiments, 200 nM PMA, 200 µg/mL AEPS and 0.06 µM captopril were selected as the optimal concentrations for further experiments.

### 4.5. Study Protocol

HUVECs at passage 3 were cultured in 6-well plates at a density of 1 × 10^5^ cells per well. Forty-eight hours after seeding, once reaching 80% confluency, HUVECs were divided into five groups: untreated control, treatment with 200 µg/mL AEPS, treatment with 200 nM PMA, concomitant treatment with 200 nM PMA and 200 µg/mL AEPS, and concomitant treatment with 200 nM PMA and 0.06 μM captopril as the positive control. All treatments were conducted concomitantly for 24 h. HUVEC samples were then collected following the 24-h treatment for subsequent measurements.

### 4.6. Measurement of ACE, AT1R, and AT2R mRNA Expression Using Quantitative Real Time Polymerase Chain Reaction (qPCR)

The mRNA expression of ACE, AT1R, and AT2R was determined using qPCR according to a previously published protocol [30]. Total RNA from HUVECs was extracted using TRI Reagent following the manufacturer’s instructions. The purity and quantity of total RNA were measured using a spectrophotometer. The complementary DNA (cDNA) was synthesized using a QuantiNovaTM reverse transcription kit (Qiagen, Hilden, Germany) according to the kit’s protocol. Primer 3 software (http://frodo:wi.mit.edu/cgi-bin/primer3/primer3-www.cgi, accessed on 15 November 2020) was used to design the qPCR primers based on the NCBI GenBank database (Table 2). Glyceraldehyde-3-phosphate dehydrogenase (GAPDH) was used as an internal control. The qPCR analysis was performed in a Bio-Rad CFX96 cycler (Bio-Rad Laboratories, Hercules, CA, USA) with initial denaturation at 95 °C for 3 min, followed by 40 cycles of 61 °C for 30 s, 95 °C for 1 min, 55 °C for 1 min, 70 cycles of 60 °C for 10 s; and terminated by a cooling step at 4 °C. The relative mRNA expression of ACE, AT1R, and AT2R was calculated based on the 2^−∆∆CT^ method, where ∆∆CT was calculated as [(CT gene of interest − CT internal control) Sample A − (CT gene of interest − CT internal control) Sample B]. Sample A represents the treated sample, and Sample B represents the untreated control.

### 4.7. Determination of the ACE and Ang II Protein Levels

The ACE and Ang II protein levels in HUVECs were determined using Human Ang II and ACE enzyme-linked immunosorbent assay (ELISA) kits (FineTest, Wuhan, China) according to the manufacturer’s instructions. HUVEC lysates were used as samples for ACE protein measurement, whereas the culture supernatant was used as samples for Ang II protein measurement. The samples and standards were added to antibody-coated 96-well plates in duplicate. Following 90 min of incubation, a biotin-labeled detection antibody was added. The plates were washed three times before adding streptavidin-horseradish peroxidase conjugate solution. Subsequently, the 3,3’,5,5’-tetramethylbenzidine substrate was pipetted into the wells. The reaction was stopped after 30 min by adding the stop solution. The optical density of each well was then measured at 450 nm using a microplate reader. The ACE and Ang II standard calibration curves were generated based on the optical density. Subsequently, the ACE and Ang II protein concentrations in the HUVEC samples were determined using these standard calibration curves.

### 4.8. Ex Vivo Aortic Ring Assay

Animal sacrifice and tissue harvesting were carried out following the protocols established by the Universiti Kebangsaan Malaysia Animal Ethics Committee. Adult Sprague Dawley rats, weighing around 250 g, were euthanized by intravenous injection of ketamine and xylazine cocktail (0.2 mL/kg). The thoracic aorta was carefully harvested, cleaned of connective tissue and fat, and cut into 2–3 mm segments, ensuring the endothelium remained intact. The rings were mounted on a wire myograph (Danish Myo Technology, Ann Arbor, MI, USA) under optimal tension (9.8 mN) and allowed to stabilize for 30 min. The vessels were immersed in Krebs solution at 37 °C and continuously aerated with 95% O_2_ and 5% CO_2_ [52]. Some of the rings were pretreated with 100 mg/mL AEPS or 0.5 µg/mL captopril for 1 h. The contraction of each prepared aorta was induced by the administration of 40 mM KCl and recorded. The cumulative contraction responses of the aortic rings to 10^−9^–10^−6^ M PMA were then measured. The PowerLab Data Acquisition System (ADInstruments, Bella Vista, Australia) measured and recorded the changes in vessel tension. Aortic contraction data were expressed as the percentage increase in KCl-induced contraction.

### 4.9. Statistical Analysis

Statistical Package for Social Sciences (SPSS) version 20.0 was used to analyze the data. All data sets were tested for normal distribution using the Shapiro–Wilk test and were normally distributed. The data were expressed as mean ± standard error for mean (SEM). The differences between the groups were analyzed using a one-way analysis of variance (ANOVA) with the post hoc Tukey test. A value of *p* < 0.05 was considered significant.

## 5. Conclusions

The current study demonstrates that AEPS inhibits the ACE/AT1R pathway by reducing ACE mRNA, protein, and activity, as well as Ang II level and AT1R expression in PMA-induced endothelial cells. These findings underscore the potential antihypertensive mechanism of AEPS. However, additional research involving the isolation of bioactive components from AEPS, clinical trials, and pharmacokinetic studies are necessary to further substantiate the therapeutic potential of AEPS for hypertension.

## Figures and Tables

**Table 1:** Compounds identified in the aqueous extract of *P. sarmentosum* leaves using the Q Exactive HF Orbitrap mass spectrometry analysis.

No.	Retention Time (min)	*m/z*	Molecular Weight	Molecular Formula	Proposed Compound	Class
1	0.70	755.20	756.21	C_33_H_40_O_20_	Quercetin 3-rhamninoside	Flavonoid
2	3.14	167.03	166.02	C_8_H_6_O_4_	Piperonylic acid	Organic acid
3	3.58	225.08	224.07	C_10_H_12_N_2_O_4_	2,4,5-Trimethoxybenzaldehyde hydrazone N-oxide	Hydrazone
4	3.65	304.11	303.11	C_16_H_17_NO_5_	Cenocladamide	Alkaloid
5	3.81	288.12	287.11	C_16_H_17_NO_4_	Pipermethystine	Alkaloid
6	4.54	200.10	199.09	C_13_H_13_NO	N-(3-Phenylpropanoyl) pyrrole	Alkaloid
7	5.05	300.15	299.15	C_18_H_21_NO_3_	1-[7-(3,4-Methylenedioxyphenyl)-2,6-heptadienoyl] pyrrolidine	Alkaloid
8	5.22	286.14	285.13	C_17_H_19_NO_3_	Piperine	Alkaloid
9	6.27	216.10	215.09	C_13_H_13_NO_2_	Sarmentamide A	Amide
10	8.16	318.13	317.12	C_17_H_19_NO_5_	Sarmentamide B	Amide
11	8.43	274.14	273.13	C_16_H_19_NO_3_	Piperlonguminine	Alkaloid
12	8.61	232.13	231.12	C_14_H_17_NO_2_	Awaine	Alkaloid
13	8.71	318.13	317.12	C_17_H_19_NO_5_	Piplartine	Alkaloid
14	8.78	306.13	305.12	C_16_H_19_NO_5_	Peepuloidine	Alkaloid
15	9.500	306.07	305.06	C_18_H_11_NO_4_	Cepharadione A	Alkaloid
16	11.53	222.18	221.17	C_14_H_23_NO	Sarmentine	Alkaloid
17	11.68	326.17	325.16	C_20_H_23_NO_3_	1-[9-(3,4-Methylenedioxyphenyl)-2,4,8-nonatrienoyl] pyrrolidine	Alkaloid
18	12.04	328.18	327.18	C_20_H_25_NO_3_	Retrofractamide	Amide

**Table 2:** Primers for qPCR amplification.

Gene	GenBank Accession No.	Type	Sequence
Angiotensin-converting enzyme (ACE)	NM_000789	Forward Reverse	5′-atg tag atg cag ggg act cg-3′ 5′-agg gca cca cca agt cat ag-3′
Angiotensin II type 1 receptor (AT1R)	NM_032049	Forward Reverse	5′-gca caa tgc ttg tag cca aa-3′ 5′-ggg ttg aat ttt ggg act ca-3′
Angiotensin II type 2 receptor (AT2R)	NM_000686	Forward Reverse	5′-ttc cct tcc atg ttc tga cc-3′ 5′-aaa cac act gcg gag ctt ct-3′
Glyceraldehyde-3-phosphate dehydrogenase (GAPDH)	NM_002046	Forward Reverse	5′-tcc ctg agc tga acg gga ag-3′ 5′-gga gga gtg ggt gtc gct gt-3′

## Data Availability

The data of this study is contained within the article.
